# A new potential energy surface for the H_2_S system and dynamics study on the S(^1^D) + H_2_(X^1^Σ_g_^+^) reaction

**DOI:** 10.1038/srep14594

**Published:** 2015-10-05

**Authors:** Jiuchuang Yuan, Di He, Maodu Chen

**Affiliations:** 1 Key Laboratory of Materials Modification by Laser, Electron, and Ion Beams (Ministry of Education), School of Physics and Optoelectronic Technology, Dalian University of Technology, Dalian 116024, PR China

## Abstract

We constructed a new global potential energy surface (PES) for the electronic ground state (^1^A′) of H_2_S based on 21,300 accurate *ab initio* energy points over a large configuration space. The *ab initio* energies are obtained from multireference configuration interaction calculations with a Davidson correction using basis sets of quadruple zeta quality. The neural network method is applied to fit the PES, and the root mean square error of fitting is small (1.68 meV). Time-dependent wave packet studies for the S(^1^D) + H_2_(X^1^Σ_g_^+^) → H(^2^S) + SH(X^2^Π) reaction on the new PES are conducted to study the reaction dynamics. The calculated integral cross sections decrease with increasing collision energy and remain fairly constant within the high collision energy range. Both forward and backward scatterings can be observed as expected for a barrierless reaction with a deep well on the PES. The calculated integral cross sections and differential cross sections are in good agreement with the experimental results.

In recent years, a flurry of research has been performed on sulfur chemistry due to its major role in environmental issues such as acid rain and air pollution. Furthermore, the S(^1^D) + H_2_(X^1^Σ_g_^+^) → H(^2^S) + SH(X^2^Π) reaction and its isotopic variants have received considerable interest as prototypes for the insertion reaction, which is often characterized by a deep potential well along the reaction path.

The S(^1^D) + H_2_(X^1^Σ_g_^+^) → H(^2^S) + SH(X^2^Π) reactive system and its isotopic variants have been studied both experimentally^1^,^2^,^3^,^4^,^5^,^6^,^7^ and theoretically^8^,^9^,^10^,^11^,^12^,^13^,^14^,^15^,^16^,^17^,^18^,^19^,^20^,^21^,^22^,^23^,^24^,^25^,^26^. Several experimental studies have been performed to accurately describe the equilibrium geometry and a local potential energy surface^1^,^2^,^3^. The integral cross sections (ICSs) and state-resolved differential cross sections (DCSs) of the S(^1^D) + H_2_ reaction were measured using crossed molecular beam experiments and a Doppler-selected time-of-flight technique^4^,^5^. For the calculations of reactive dynamics, potential energy surface (PES) that describes the interaction in the molecular system is crucial. In recent decades, several three-dimensional ground-state (^1^A′) PESs of H_2_S system have been reported^10^,^11^,^12^,^13^. Zyubin *et al.* reported an electronic ground-state PES (Zyubin PES) using the reproducing kernel Hilbert space (RKHS) approach based on 2000 energy points calculated at the multi-reference configuration interaction (MRCI) level with multi-configuration self-consistent field (MCSCF) reference wave functions^10^. On the PES, the authors identified a barrierless insertion pathway along the T-shaped geometry and a small barrier (8 kcal/mol) along the collinear geometry. Based on the Zyubin PES, Skodje and co-workers studied the title reaction using the QCT method^8^. The results were consistent with an insertion mechanism mediated through capture dynamics in the entrance channel followed by the statistical decay of a long-lived complex. In 2002, Ho *et al.* provided a new interpolation of the *ab initio* data of Zyubin *et al.* and constructed a global PES (Ho PES) within the framework of RKHS formalism^11^. Since the Ho PES was reported, several dynamics studies^16^,^17^,^18^,^21^,^23^ have been conducted using the PES. Honvault *et al.*^23^ studied the title reaction using accurate three-dimensional quantum-mechanical (QM) scattering method at a collision energy of 2.24 kcal/mol. They found that the DCS result exhibits forward/backward symmetry, which is characteristic of an insertion reaction. Later, Banares *et al.*^16^ conducted QM and QCT calculations at collision energies of 2.24 kcal/mol and 3.96 kcal/mol, and the theoretical results were used to simulate the experimental data. Based on Ho PES, the isotope effect of the title reaction was studied^18^,^21^ using QM and QCT methods. In 2009, Song *et al.* reported a realistic global PES with the double many-body expansion (DMBE) method, which is widely used to construct PESs^27^,^28^,^29^, based on 1984 energy points calculated at the MRCI level using the full valence complete active space (FVCAS) reference function^12^. The PES is referred to as the DMBE/CBS PES. The intramolecular isotope effect obtained by exploratory dynamics calculations on the DMBE PES reproduces the experimental result^12^. In the same year, Song *et al.* provided another PES for this reactive system using a DMBE-scaled external correlation (SEC) method based on 1972 energy points^13^, with the PES is referred to as the DMBE/SEC PES. Hankel *et al.*^20^ presented exact quantum ICSs and DCS for the title reaction using DMBE/CBS PES and DMBE/SEC PES, and they found that the results obtained on the DMBE/CBS PES were smaller due to an unphysical barrier. The influence of the barrier on the DMBE/CBS PES at low energies was investigated^9^ in detail through a comparion with the results obtained on the Ho PES, and the two dynamical behaviors were very different. Jambrina *et al.*^22^ calculated the cumulative reaction probabilities of the title reaction using three different theoretical approaches: time-independent QM, QCT and statistical QCT. The agreement among the three methods was good indicating that the title reaction can be considered statistical.

The reaction of S(^1^D) + H_2_(X^1^Σ_g_^+^) → H(^2^S) + SH(X^2^Π) can occur within the very low collision energy range due to exothermicity and the absence of a barrier along the reactive path. Recently, reaction dynamics in the low temperature regime have attracted a great deal of attention, because the information on reactive processes within the very low kinetic energy range can be explored by newly developed experimental techniques. In 2010, Berteloite *et al.* reported the absolute rate coefficients down to 5.8 K and ICSs to the collision energy as low as 0.68 meV obtained by kinetics and crossed-beam experiments, and they found that the cumulative contribution of various partial waves can explain the behavior of the excitation function^6^. In 2012, the ICSs for the two channels of the S(^1^D) + HD reaction were obtained through crossed-beam experiments down to 0.46 meV.^7^ On the reactive path of the S(^1^D) + H_2_(X^1^Σ_g_^+^) → H(^2^S) + SH(X^2^Π) reaction, there is a deep and wide well in which the long-life complex can form at a low collision energy; thus, it can be expected that the dynamics results are extremely sensitive to the PES. Therefore, the accuracy of the PES is crucial for dynamics calculations of the title reaction because significant error in reaction dynamics may arise from a small inaccuracy on the PES. Moreover, for the barrierless reaction, the long-range interaction plays an important role, especially within the very low collision energy range. Hence, a correct description of the potential energy in the long-range region is of great importance to the dynamics calculation. In this work, our major goal is to construct an accurate global PES for the H_2_S (^1^A′) system, which can meet the requirements above. To achieve this objective, we calculated a mass of *ab initio* energy points in a large region of configuration space, and used the neural network (NN) method to fit the new PES. Then, time-dependent wave packet (TDWP) calculations were conducted for the S(^1^D) + H_2_(X^1^Σ_g_^+^) → H(^2^S) + SH(X^2^Π) reaction to study the reactive mechanism and verify the new PES.

## Results

### Characteristics of the potential energy surface

**Figure 1:**
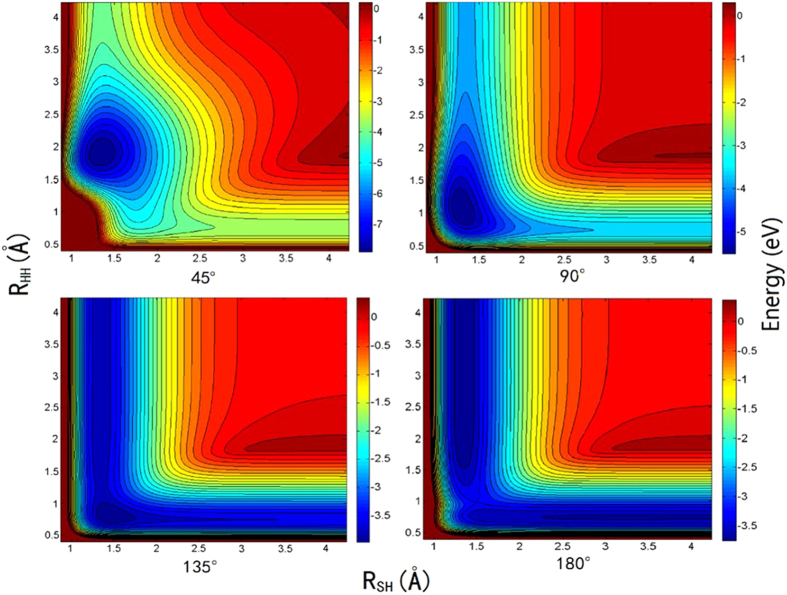
Potential energy surfaces of the H_2_S (^1^A′) system for the four S-H-H angles 45º, 90º, 135º and 180º.

**Figure 2:**
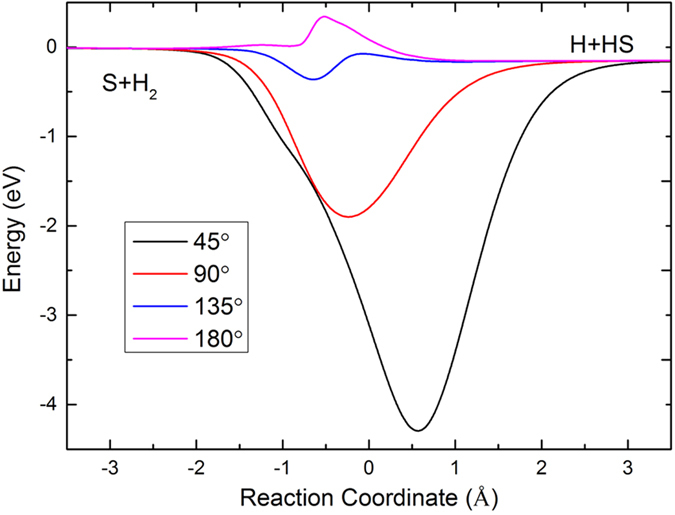
Minimum energy paths for H_2_S (^1^A′) PES at four S-H-H angles.

**Figure 3:**
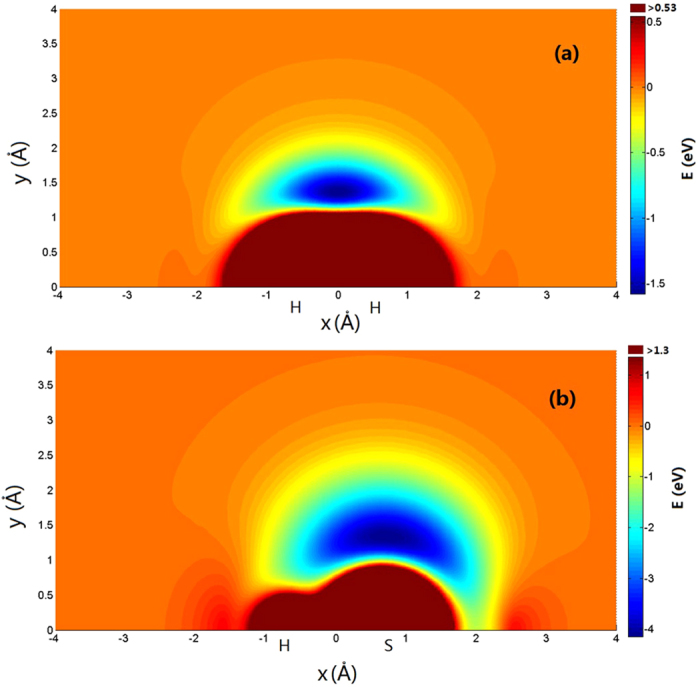
(**a**) Color plot for the S(^1^D) atom moving around the H_2_(X^1^Σ_g_^+^) molecule, of which the bond length is fixed at the equilibrium distance. (**b**) Color plot for the H(^2^S) atom moving around the SH(X^2^Π) molecule, of which the bond length is fixed at the equilibrium distance.

Table 1 shows the equilibrium structural parameters of the new H_2_S PES. The experimental results^30^ and the previous theoretical results from two analytical PESs^12^,^13^ are also listed in the table. For both the geometry and the energy, the agreement between the calculated and experimental results is good, and our results are closer to the experimental data. The contour maps for the S(^1^D) + H_2_(X^1^Σ_g_^+^) → H(^2^S) + SH(X^2^Π) reaction in internal coordinates at four S-H-H angles (45°, 90°, 135°, and 180°) are shown in Figure 1. In each map, there are two valleys: the left valley corresponds H(^2^S) + SH(X^2^Π), and the valley at the bottom corresponds to S(^1^D) + H_2_(X^1^Σ_g_^+^). There are wells at three angles (45°, 90° and 135°), and the wells are deeper for larger angles. For the angle of 180°, there is no well but there is a barrier, indicating that the title reaction with a sufficiently low collision energy cannot occur through the S-H-H collinear path. For a better understanding of the characteristics of the PES, the minimum energy paths (MEPs) of the S(^1^D) + H_2_(X^1^Σ_g_^+^) → H(^2^S) + SH(X^2^Π) reaction at the four S-H-H angles are shown in Fig. 2. The exothermicity for the S(^1^D) + H_2_(X^1^Σ_g_^+^) → H(^2^S) + SH(X^2^Π) reaction is 0.15 eV. For the S-H-H angles (45°, 90° and 135°), the depths of the wells are 0.36 eV, 1.90 eV and 4.29 eV, respectively. For the S-H-H angle of 180°, the barrier height is 0.34 eV. Figure 3(a) shows an energy plot for a sulfur atom moving around a H_2_ molecule with a fixed bond length at the equilibrium distance. The zero energy is set as the energy in the configuration when the sulfur atom is far from the H_2_ molecule. There is a well 1.58 eV deep at x = 0.0 angstrom, y = 1.37 angstrom. For the S(^1^D) + H_2_(X^1^Σ_g_^+^) → H(^2^S) + SH(X^2^Π) reaction, as the sulfur atom moves slowly to the H_2_ molecular, it is attracted to the well. Therefore, it is expected that the insertion reaction plays an important role in the title reaction. Figure 3(b) shows an energy plot for a hydrogen atom moving around a SH molecule of which the bond length is fixed at the equilibrium distance and the zero energy is set as the energy in the configuration when the hydrogen atom is far from the SH molecule. There is a deep well (4.15 eV) close to the sulfur atom at of x = 0.74 angstrom, y = 1.33 angstrom.

### Molecular reaction dynamics

**Figure 4:**
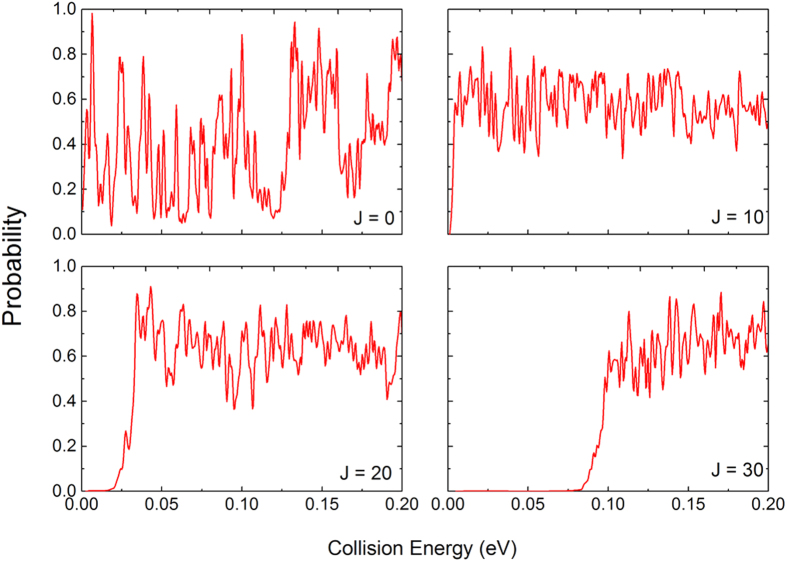
Total reaction probabilities of the S(^1^D) + H_2_(X^1^Σ_g_^+^) → H(^2^S) + SH(X^2^Π) reaction calculated by the TDWP method as a function of the collision energy for the total angular momentum J = 0, 10, 20 and 30.

Figure 4 presents the reaction probabilities (J = 0, 10, 20 and 30) of the S(^1^D) + H_2_(X^1^Σ_g_^+^) → H(^2^S) + SH(X^2^Π) reaction calculated by the TDWP method as a function of the collision energy. As shown in Fig. 4, a large number of sharp and dense oscillations are found in the reaction probabilities, especially at low collision energies. The S(^1^D) + H_2_(X^1^Σ_g_^+^) → H(^2^S) + SH(X^2^Π) reaction is a typical insertion reaction characterized by a deep well that can support a larger number of bound and quasibound states. The deep well on the H_2_S PES and small exoergicity of the title reaction are the major causes of the highly oscillatory feature. The resonance forms in the deep well. Because of the small exoergicity of the title reaction, there are few available product channels for the resonances to decay into. Therefore, long-lived resonances are expected in the title reaction, which leads to the strong oscillations in the reaction probabilities. This explanation can be validated by other insertion reactions. The insertion reaction of C(^1^D) + H_2_^31^, which also has a deep well and a small exoergicity, has similar highly oscillatory feature. However, for the insertion reactions^32^ of N(^2^D) + H_2_ and O(^1^D) + H_2_, there are several broad peaks in the reaction probabilities indicating short-lived resonances, which is different from the title reaction. Because the insertion reactions N(^2^D) + H_2_ and O(^1^D) + H_2_ have greater exoergicities, and there are more available product channels for the resonances to decay into. For J = 0, the reaction probabilities do not exhibit an energy threshold because there is a small exoergicity for the S(^1^D) + H_2_(X^1^Σ_g_^+^) → H(^2^S) + SH(X^2^Π) reaction and no barrier on the reactive path. For high values of J (10, 20 and 30), the energy threshold can be found in the reaction probabilities and increases with an increasing J value, which can be attributed to the centrifugal barrier that increases with the increase in the J value.

**Figure 5:**
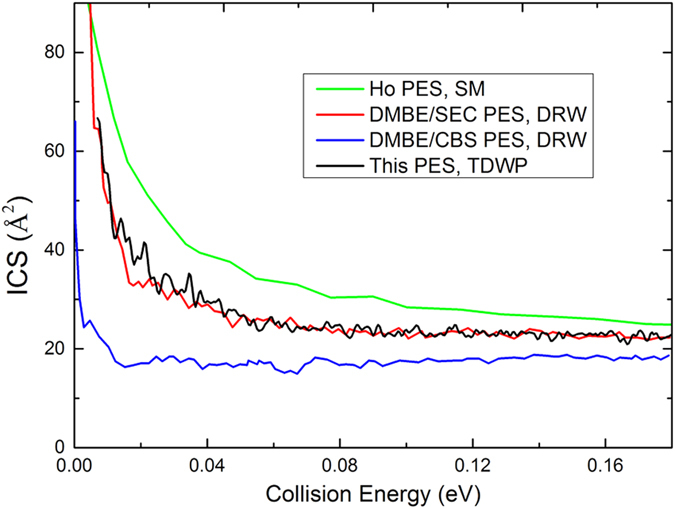
The total ICS of the S(^1^D) + H_2_(X^1^Σ_g_^+^) → H(^2^S) + SH(X^2^Π) reaction calculated by the TDWP method. Also included are the theoretical results^21^ from SM calculations employing the Ho PES and the theoretical results^20^ obtained from the DRW code on DMBE/SEC PES and DMBE/CBS PES.

**Figure 6:**
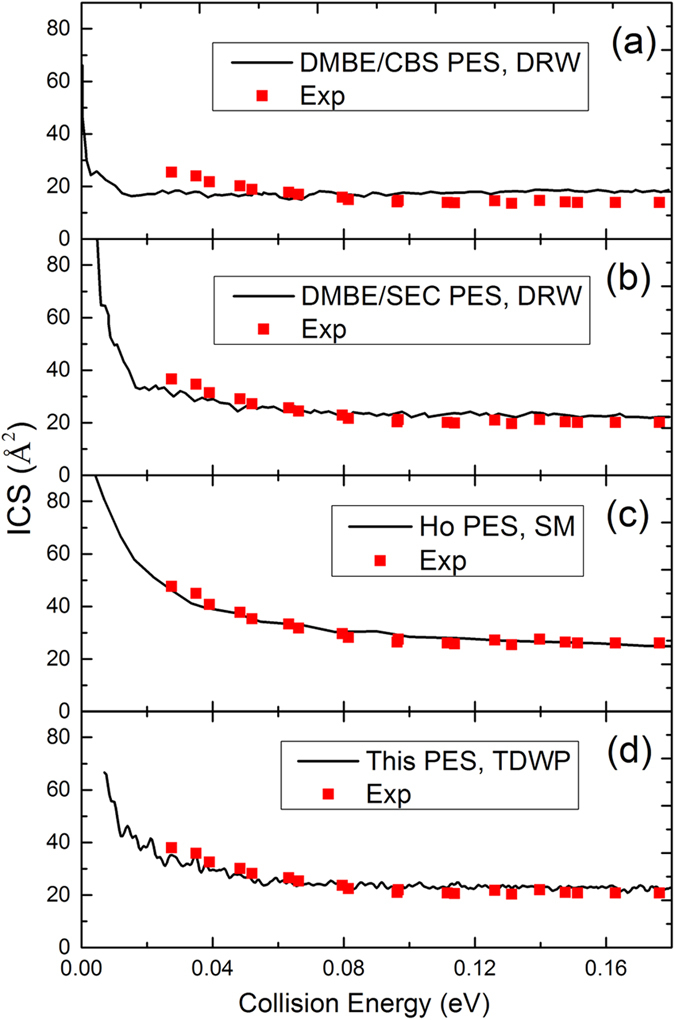
The theoretical ICSs of the S(^1^D) + H_2_(X^1^Σ_g_^+^) → H(^2^S) + SH(X^2^Π) reaction compared with the experimental data^4^. The previous theoretical results include the ICSs^20^ calculated using the DRW code on DMBE/CBS PES (**a**) and DMBE/SEC PES (**b**) and ICSs^21^ obtained from SM calculations employing the Ho PES (**c**). Our results (**d**) were obtained using the TDWP method on the new PES. The experimental results have been scaled to match each of the theoretical results. The scale coefficient minimizes the RMSE between the experimental data and the corresponding theoretical results.

In the TDWP calculation, the maximum total angular momentum quantum number is 45, and the threshold energy for J = 45 is above 0.2 eV. Therefore, the collision energy of 0.2 eV is set as the upper limit of the collision energy in the calculations of the ICSs. Figure 5 shows the total ICS of the S(^1^D) + H_2_(X^1^Σ_g_^+^) → H(^2^S) + SH(X^2^Π) reaction calculated by the TDWP method. Within the very low collision energy range, the calculated ICSs have high values and decrease rapidly with increasing collision energy, which is a typical feature of exothermic reactions without a barrier. With increasing collision energy, the rate of decrease becomes lower and the values of the ICSs remain fairly constant within the high collision energy range. Oscillations are present on the curve of the calculated ICSs, which is a sign of quantum resonance. For the H_2_S system, there is a sufficiently deep potential well on the PES to support a mass of quantum states, which leads to the resonance effect. For comparison, three previous results are also shown in Fig. 5: the theoretical results^21^ from the statistical model (SM) calculations employing the Ho PES, and the theoretical results^20^ obtained from the DIFFREALWAVE (DRW) code on DMBE/SEC PES and DMBE/CBS PES. The ICSs for DMBE/CBS PES are much smaller than the results obtained from other PESs because of an unphysical barrier for contracted H_2_ distances^20^. Figure 5 also shows that the Ho PES results are the largest in these theoretical results, and Hankel *et al.* believed that the shallow well present on the Ho PES causes this feature. The ICSs obtained from DMBE/SEC PES are very similar to our results, except that our results are larger within the energy range from 0.012 eV to 0.04 eV. To verify the accuracy of the new PES, we compare the theoretical results with experimental data reported by Liu and co-workers^4^ in Fig. 6. The unit in the measurement is an arbitrary unit (a.u.); thus, it is necessary to scale the experimental results to each of the theoretical results. Each scale coefficient is determined to minimize the root mean square error (RMSE) between the experimental data and the corresponding theoretical results. As shown in the figure, the agreement between our calculated results and the experimental results is strikingly good for the range of the collision energy, and the ICSs obtained from the Ho PES also match the experimental data. For the DMBE/CBS PES, the results are too flat to reproduce the experimental results. The DMBE/SEC PES results agree with the measurement results at high collision energy, but at low collision energy (the first two data points), the calculated values are significantly smaller than the experimental results.

**Figure 7:**
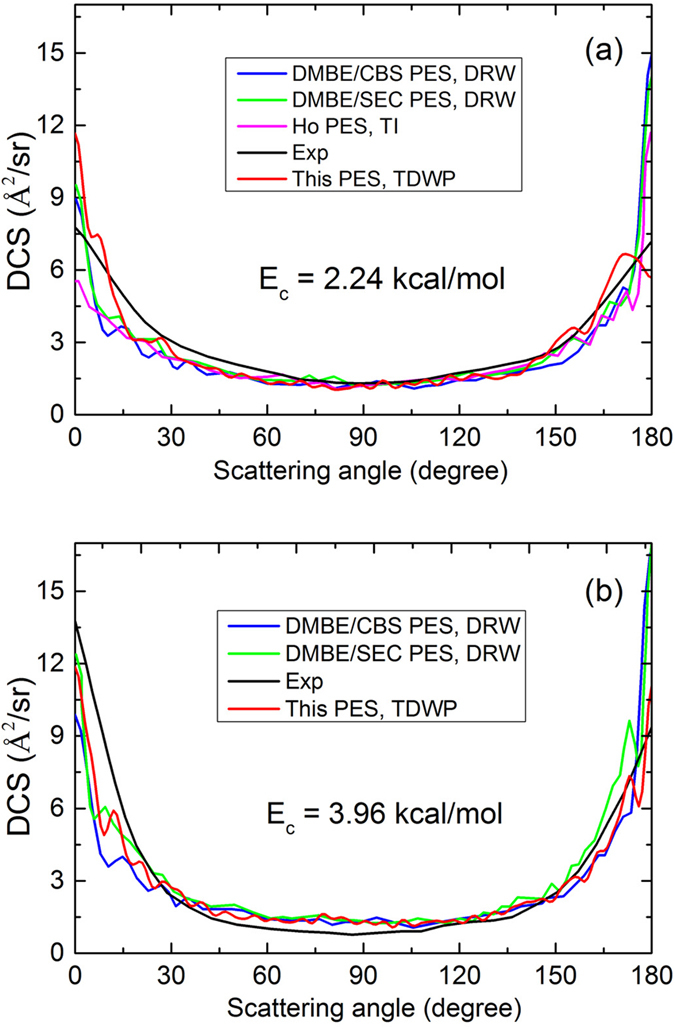
The total DCSs of the S(^1^D) + H_2_(X^1^Σ_g_^+^) → H(^2^S) + SH(X^2^Π) reaction calculated by the TDWP method at two collision energies ((a) 2.24 kcal/mol and (b) 3.96 kcal/mol), compared with the experimental data 5 and previous theoretical results^16^,^20^. Because the theoretical results^20^ obtained from DMBE/CBS and DMBE/SEC PESs are relative, previous theoretical results have been scaled separately to make the value at 90º equal to our result.

To further verify the new PES, we calculated the total DCSs of the S(^1^D) + H_2_(X^1^Σ_g_^+^) → H(^2^S) + SH(X^2^Π) reaction according to the TDWP method at the two collision energies (2.24 kcal/mol and 3.96 kcal/mol) displayed in Fig. 7. The experimental results^5^ and several theoretical results^16^,^20^ are also shown in the figure. Because the theoretical results obtained from DMBE/CBS and DMBE/SEC PESs are relative in the paper of Hankel and co-workers^20^, the previous theoretical results have been scaled separately to make the value at 90° equal to our result for comparison. For the experimental results and all of the theoretical results, both forward and backward scattering can be observed in the reaction as expected for a reaction with a deep well and no barrier. The DCSs are not strictly backward-forward symmetric. At the collision energy of 2.24 kcal/mol, a slight forward preference is found in the experimental results, and the DCSs obtained from the new PES reproduce this feature well. However, other previous theoretical results, particularly the DCSs^16^ obtained from Ho PES, show an opposite preference. For the collision energy of 3.96 kcal/mol, the forward preference in the experiment results is more significant, and our results match the preference well. The DCSs obtained from DMBE/CBS and DMBE/SEC PESs show a significant backward preference, contrary to the experimental results.

## Discussion

In this work, we report a new global PES for the electronic ground state of the H_2_S system. A mass of *ab initio* energies over a large configuration space are calculated at the MRCI level with the aug-cc-pVQZ basis sets plus the Davidson correction. The diatomic potential and the three-body term are fitted by the NN method. For the diatomic potential, the precision of the fitting is extremely high, and the RMSEs are 5.1 × 10^−5^ eV for 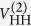 and 1.5 × 10^−5^ eV for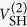. The overall RMSE of the new PES is only 1.68 meV. For the equilibrium structural parameters of the H_2_S system, the agreement between the calculated and the experimental results is good, and our results are closer to the experimental data than those obtained from two previous analytical PESs^12^,^13^. Based on the new PES, the TDWP calculation is performed for theS(^1^D) + H_2_(X^1^Σ_g_^+^) → H(^2^S) + SH(X^2^Π) reaction. The reaction probabilities, ICSs and DCSs of the title reaction are obtained. A large number of sharp oscillations are found in the reaction probabilities, especially at low collision energies. The deep well on the H_2_S PES and small exoergicity of the title reaction are the major causes of the highly oscillatory feature. To further verify the new PES, we have compared our dynamics results (ICSs and DCSs) with experimental^4^,^5^ and theoretical results^16^,^20^,^21^. The ICSs obtained from our new PES agree with the experimental results^4^ for the entire range of collision energy. The results^20^ from two DMBE PESs do not match the experimental data exactly. The ICSs^21^ from the Ho PES reproduce the experimental data well, but the DCSs^16^ obtained from Ho PES at the collision energy of 2.24 kcal/mol show a significant backward preference, contrary to the experimental results. In the experimental results, a slight forward preference is found at the collision energy of 2.24 kcal/mol, and the forward preference becomes more significant at the collision energy of 3.96 kcal/mol. The DCSs on our new PES reproduce this forward preference. The close agreement of our results with the experimental data shows that the new PES is sufficiently accurate to describe the dynamics of the title reaction. In addition to the high accuracy, another advantage of the new PES is the large configuration space employed in the calculations for the *ab initio* energy points. The points for our new PES extend to larger distances (18.5 Å) than other previous PESs (less than 6.5 Å). Thus, the new PES provides a good description of not only short-range regions but also long-range regions. The title reaction is barrierless and exothermic; thus, the interaction within the long-range regions is important, especially in a low-temperature regime. Based on the qualities of the new PES described above, we believe that the new PES provides a good description of the reaction dynamics in the low-temperature regime, and additional research will be conducted in the future.

## Methods

### Potential Energy Surface

#### *Ab initio* calculations

The *ab initio* calculations were conducted at the MRCI level with the CASSCF reference wave functions with the MOLPRO package^33^. The aug-cc-pVQZ basis set of Dunning was employed for H and S atoms. In the arrangement channel S(^1^D) + H_2_, the supermolecules are correlated with three states (1A′, 2A′, 1A″). Therefore, all of the three states were assigned equal weight factors in the state-averaged complete active space self-consistent field (SA-CASSCF) calculation. The wave function of SA-CASSCF was used in the internally contracted MRCI method as a reference. In addition, the higher excitations were included using a multi-reference Davidson correction (+Q). Eight valence electrons were in 10 active orbitals (9a′ + 1a″) composed of two 1s orbitals on H and 2s, 2p, 3s and 3p orbitals on S. Furthermore, the remaining orbitals were doubly occupied in all *ab initio* calculations. To achieve a high-accuracy PES, a large number of configurations (~21,300) are chosen to calculate the *ab initio* energy. Due to the importance of the long-range potential at the low collision energy range, a large region of configuration space is applied in the calculation: the S-H_2_ region is defined by 0.37 ≤ R_HH_/Å ≤ 18.5, 0 ≤ R_S-HH_/Å ≤ 18.5, 0 ≤ *θ/*degree ≤ 90, and the H-SH region is defined by 0.79 ≤ R_SH_/Å ≤ 18.5, 0 ≤ R_H-SH_/Å ≤ 18.5, 0 ≤ *θ/*degree ≤180. For the diatomic potential of H_2_ and SH, 141 and 146 points are calculated using the method described above, respectively.

### Fitting the potential energy surface

The global analytical surface of the triatomic system can be presented as


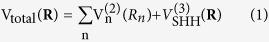


where **R** is a collective variable of all internuclear distances, 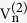 (n = HH, SH_a_, SH_b_) is the diatomic potential, *R*_n_ is the distance between two atoms, and 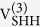 is the three-body term. In this work, the diatomic potential and the three-body term are fitted by the NN^34^ method, which is inspired by the central nervous system of animals. The basic unit of NN is the neuron, and as a synapse, the neuron receives input signals and emits an output signal. The output signal y can be written as follows:


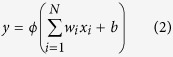


where *x*_*i*_ (*i *= 1, …, N) is the input signals, *w*_*i*_ is the connection weight, *b* is a bias, and *ϕ* is a transfer function. The structure of the NN is important to the computational efficiency and fitting precision; thus, we used a series of tests to determine the structures. For the two-body potential of HH, two hidden layers are included in the NN, and there are six neurons in each neuron. Two hidden layers are used to fit the different parts of the SH two-body potential, and the structures of the hidden layers are (5, 5) and (4, 4), respectively. The precision of the fitting is extremely high and the RMSEs are 5.1 × 10^−5^ eV for 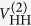 and 1.5 × 10^−5^ eV for 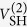.

For the S(^1^D) + H_2_(X^1^Σ_g_^+^) → H(^2^S) + SH(X^2^Π) reaction, the product molecule SH(X^2^Π) dissociates to the ground-state atoms S(^3^P) and H(^2^S). Due to the transition of the state of the sulfur atom, the energy changes dramatically at certain configurations, forming cusp structures. Most of these cusp structures are far from the strong interaction region, and the energies near cusp structures are relatively high. Therefore, the region near the cusp structures has a slight impact on the calculation of the reaction dynamics, especially at the low collision energy range. However, cusp structures cause troubles in the fitting process because the fitting errors near cusp structures are relatively large, which may reduce the accuracy of the PES. To solve this problem, Zyubin *et al.*^10^ used an anti-cusp to compensate, whereas Song *et al.* introduced a switching function^12^,^13^. In the present work, the configuration space is divided into two parts. Most of the configurations with relatively low energy exist in one part named Part 1, and most of the cusp structures exist in another part named Part 2. The NN fitting is performed for the two parts separately. With the divided method, the fitting precision improves greatly for Part 1 and Part 2. The high fitting precision for Part 1 is important for the calculation of the reaction dynamics, especially at low collision energies because most of the interaction regions are included in Part 1. The low-order permutation invariant polynomials (PIPs)^35^,^36^ are applied in the fitting process to solve the problem of the adaptation of the permutation symmetry. For each part, more than twenty NN PESs are obtained, and several NN PESs (seven NN PESs for Part 1 and four NN PESs for Part 2) with the least fitting errors are selected to structure an average PES. The overall RMSE of the average PES is small (only 1.68 meV).

### Dynamical Calculations

Based on the new PES, the dynamics of the S(^1^D) + H_2_(X^1^Σ_g_^+^) → H(^2^S) + SH(X^2^Π) reaction are studied by the TDWP method. The TDWP method is a powerful tool with which to study reaction dynamics and has been applied in our previous studies^37^,^38^,^39^. In this paper, the TDWP method is introduced in brief, and more details can be found in the relevant literature^40^,^41^. In the body fixed representation, the reactant Jacobi coordinates are used. The Hamiltonian can be written as


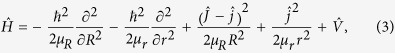


where *R* and *r* are defined as the distances of S-H_2_ and H-H, respectively. *μ*_*R*_ and *μ*_*r*_ are the reduced masses associated with *R* and *r* coordinates, respectively. 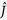 is the total angular momentum operator, and 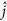 is the angular momentum operator of reactant diatom molecule H_2_. 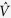 is the potential energy of the H_2_S system. The reactant coordinate based method^42^ is used to extract the state-to-state S-matrix. The state-to-state reaction probability can be obtained by


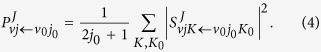


The state-to-state ICSs and DCSs are calculated by


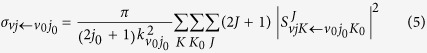


and


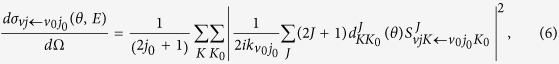


in which *θ* is the scattering angle. In this work, the ground rovibrational state (v_0_ = 0, j_0_ = 0) of the reactant is considered. Numerous tests were conducted to determine the optimal numerical parameters shown in Table 2.

## Additional Information

**How to cite this article**: Yuan, J. *et al.* A new potential energy surface for the H_2_S system and dynamics study on the S(^1^D) + H_2_(X^1^Σ_g_^+^) reaction. *Sci. Rep.* **5**, 14594; doi: 10.1038/srep14594 (2015).

## Figures and Tables

**Table 1:** Equilibrium structural parameters of the H_2_S PES.

	**∠H-S-H (degree)**	**R_SH_(Å)**	**Δ*V***^ d^**(kcal/mol)**
DMBE/CBS^a^	92.6	1.340	−99.30
DMBE/SEC^b^	92.8	1.339	−99.04
This work	92.6	1.339	−99.15
Experiment^c^	92.2	1.328	−99.10

^a^Calculated using DMBE/CBS PES^12^.

^b^Calculated using DMBE/SEC PES^13^.

^c^Experiment values^30^.

^d^Relative to the S(^1^D) + H_2_(X^1^Σ_g_^+^) asymptote.

**Table 2:** Numerical parameters used in the TDWP calculations.

**S(^1^D) + H_2_(X^1^Σ_g_^+^) → H(^2^S) + SH(X^2^Π)**	
Grid/basis range and size	R 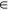 [0.005 Å, 17.5 Å], 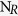 = 389r 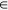 [0.005 Å, 17.5 Å], 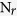 = 389 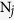 = 99
Initial wave packet 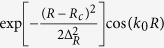	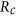 = 10.6 Å 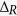 = 0.159 Å 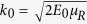 with 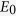 = 0.12 eV
Total propagation time	200000 iterations
Highest J value	45
